# Unusual Causes of Plumbism

**Published:** 1916-07

**Authors:** 


**Unusual Causes of Plumbism.** Hall, Lancet, Oct. 2, 1915, mentions a case due to habitual use of a soft drink which was conducted from the cellar through a lead pipe and yielded 1 grain of lead per gallon. Another case was due to the habit of carrying shot in the vest pocket, for an air gun used for cats. Fine lead dust was present in the pocket. (Note. This is appropriated from the Boston Med. & Surg. Jour. We did not suppose that this journal would thus translate the word waistcoat and it seems incredible that the word vest was used in the original. Also, what is the English of soft drink?)

